# Effects of body habitus on contrast-induced acute kidney injury after percutaneous coronary intervention

**DOI:** 10.1371/journal.pone.0203352

**Published:** 2018-09-13

**Authors:** Toshiki Kuno, Yohei Numasawa, Mitsuaki Sawano, Toshiomi Katsuki, Masaki Kodaira, Ikuko Ueda, Masahiro Suzuki, Shigetaka Noma, Koji Negishi, Shiro Ishikawa, Hiroaki Miyata, Keiichi Fukuda, Shun Kohsaka

**Affiliations:** 1 Department of Cardiology, Japanese Red Cross Ashikaga Hospital, Ashikaga, Japan; 2 Department of Medicine, Mount Sinai Beth Israel Medical Center, New York, NY, United States of America; 3 Department of Cardiology, Keio University School of Medicine, Tokyo, Japan; 4 Department of Cardiology, Saitama National Hospital, Wako, Japan; 5 Department of Cardiology, Saiseikai Utsunomiya Hospital, Utsunomiya, Japan; 6 Department of Cardiology, Yokohama Municipal Citizens' Hospital, Yokohama, Japan; 7 Department of Cardiology, Saitama City Hospital, Saitama, Japan; 8 Department of Health Policy and Management, Keio University, Tokyo, Japan; University of Sao Paulo Medical School, BRAZIL

## Abstract

**Background:**

Limiting the contrast volume to creatinine clearance (V/CrCl) ratio is crucial for preventing contrast-induced acute kidney injury (CI-AKI) after percutaneous coronary intervention (PCI). However, the incidence of CI-AKI and the distribution of V/CrCl ratios may vary according to patient body habitus.

**Objective:**

We aimed to identify the clinical factors predicting CI-AKI in patients with different body mass indexes (BMIs).

**Methods:**

We evaluated 8782 consecutive patients undergoing PCI and who were registered in a large Japanese database. CI-AKI was defined as an absolute serum creatinine increase of 0.3 mg/dL or a relative increase of 50%. The effect of the V/CrCl ratio relative to CI-AKI incidence was evaluated within the low- (≤25 kg/m^2^) and high- (>25 kg/m^2^) BMI groups, with a V/CrCl ratio > 3 considered to be a risk factor for CI-AKI.

**Results:**

A V/CrCl ratio > 3 was predictive of CI-AKI, regardless of BMI (low-BMI group: odds ratio [OR], 1.77 [1.42–2.21]; P < 0.001; high-BMI group: OR, 1.67 [1.22–2.29]; P = 0.001). The relationship between BMI and CI-AKI followed a reverse J-curve relationship, although baseline renal dysfunction (creatinine clearance <60 mL/min, 46.9% vs. 21.5%) and V/CrCl ratio > 3 (37.3% vs. 20.4%) were predominant in the low-BMI group. Indeed, low BMI was a significant predictor of a V/CrCl ratio > 3 (OR per unit decrease in BMI, 1.08 [1.05–1.10]; P < 0.001).

**Conclusions:**

A V/CrCl ratio > 3 was strongly associated with the occurrence of CI-AKI. Importantly, we also identified a tendency for physicians to use higher V/CrCl ratios in lean patients. Thus, recognizing this trend may provide a therapeutic target for reducing the incidence of CI-AKI.

## Introduction

Contrast-induced acute kidney injury (CI-AKI) in patients undergoing percutaneous coronary intervention (PCI) is common and is associated with an increased risk of mortality during and after hospitalization [1,2]. Considering the high mortality rate associated with CI-AKI, numerous large studies have attempted to identify factors that predict CI-AKI. Among the identified factors, minimizing contrast agent volume (CV) is a proven strategy for lowering the risk of CI-AKI [3]. Clinical guidelines, therefore, recommend reducing the contrast volume to creatinine clearance (V/CrCl) ratio during PCI [4,5]. The predicted CV, however, may vary depending on patient-specific characteristics, including the individual’s body mass index (BMI, kg/m^2^), with higher rates of CI-AKI reported in lean patients [6]. However, the V/CrCl ratio that is predictive of CI-AKI among patients with different BMIs has not been definitively determined.

Accordingly, this study investigated the relationship between BMI and CI-AKI, and quantified the V/CrCl ratio predictive of CI-AKI among patients with low and high BMIs. In addition, we investigated the association between a V/CrCl ratio less than 3 and BMI; a previous study determined that a V/CrCl ratio of 3 was a reasonable cut-off value [7]. The identification of clinical factors predictive of CI-AKI in patients with various BMIs is clinically important because they would provide potential therapeutic targets that might reduce the incidence of CI-AKI and inform clinical decisions regarding the optimal CV for a patient.

## Materials and methods

The Japanese Cardiovascular Database-Keio inter-hospital Cardiovascular Studies (JCD-KiCS) is a large, ongoing, multicenter, prospective cohort study designed to collect clinical background and outcome data for consecutive patients undergoing PCI. The Institutional Review Board of Keio University School of Medicine as well as each participating hospital approved the JCD-KiCS registry study protocol. The study was carried out in accordance with the approved guidelines and the Declaration of Helsinki. Before the launch of the registry, the objectives of the present study, its social significance, and an abstract were provided for clinical trial registration with the University hospital Medical Information Network (UMIN), which is recognized by the International Committee of Medical Journal Editors as an “acceptable registry” according to a statement issued in September 2004 (UMIN R000004736). UMIN is the largest and most versatile academic network information center for biomedical sciences in the world, and it is now considered as an indispensable information infrastructure for the Japanese medical community (http://www.umin.ac.jp/english/whatisumin.htm). Further, written informed consent was obtained from each patient before being included in the study. The data collection procedures and audit processes for the JCD-KiCS have been previously described, in detail [8–16]. The majority of the clinical variables included in the JCD-KiCS were defined according to the National Cardiovascular Data Registry (NCDR), sponsored by the American College of Cardiology [17–19].

**Fig 1:**
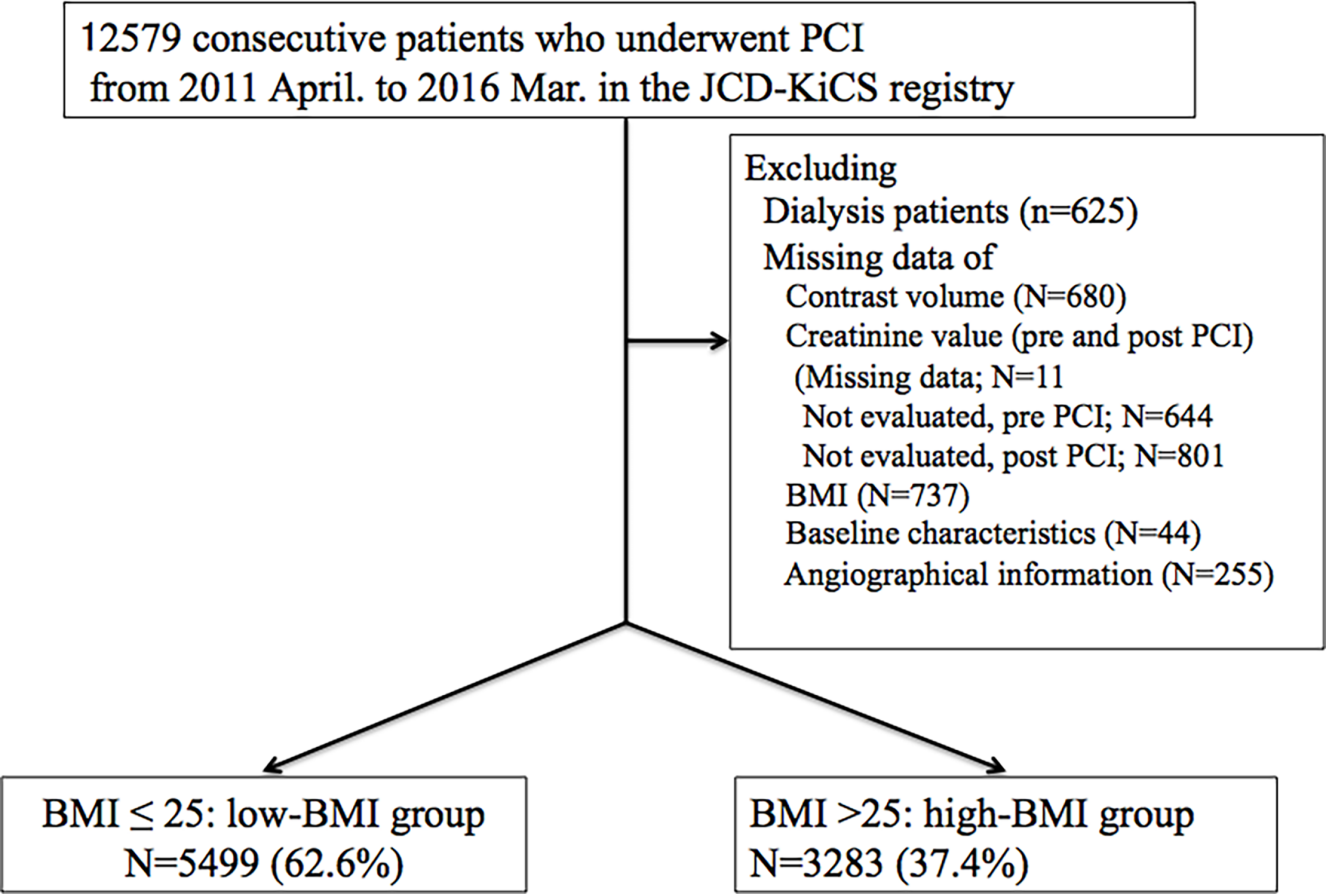
Study flow chart. PCI, percutaneous coronary intervention; JCD-KiCS, Japanese Cardiovascular Database-Keio inter-hospital Cardiovascular Studies; BMI, body mass index.

We analyzed the data for 8782 consecutive patients who underwent PCIs, between April 2011 and March 2016, across the 15 Japanese hospitals participating in the JCD-KiCS (Fig 1); patients with missing values, such as those for creatinine, CV, and BMI, were excluded. The baseline characteristics and in-hospital outcomes of the excluded patients are shown in S1 and S2 Tables.

In our registry, fifteen hospitals from the Tokyo, Tochigi, Saitama, Chiba, and Kanagawa Prefectures of Japan participated. The participating hospitals included 12 large, tertiary care, referral centers (>200 beds) and 3 mid-sized satellite hospitals (<200 beds). Among these hospitals, the average annual case volume was approximately 228. Between 2013 and 2016, we limited data registration to the 4 high-volume centers (N = 5749 among 12,579 cases), with an average case volume of 479. These numbers are not dissimilar from the median PCI cases/year across all the hospitals in Japan (median, 216; interquartile range [IQR], 121–332) [20].

The relationship between CI-AKI development and BMI was investigated, first. Then, patients were divided into 2 BMI groups: those with BMIs ≤25 kg/m^2^ (low-BMI group, n = 5499, 62.6%) and those with BMIs >25 kg/m^2^ (high-BMI group, n = 3283, 37.4%) (Fig 1). The baseline characteristics and in-hospital outcomes, including the numbers of patients developing CI-AKI, for each group were evaluated. Creatinine clearances (CrCls) were calculated using the Cockcroft-Gault formula [7,21]. In addition, to assess V/CrCl ratio variations relative to CI-AKI risk, we investigated the CI-AKI risk and mean V/CrCl ratio for all patients in both groups; our registry incorporated the validated NCDR AKI risk model [22,23].

CI-AKI was defined as an absolute increase in serum creatinine values of 0.3 mg/dL or a relative increase of 50%, in accordance with the Acute Kidney Injury Network criteria [23,24]. In the JCD-KiCS registry, the post-procedural creatinine values were defined as the highest values within 30 days after the index procedure. Congruent with the NCDR definition, if more than 1 post-procedural creatinine level was measured, the highest value was used for determining CI-AKI. The timing of the creatinine measurements in the core hospital center (Keio University Hospital; n = 979) showed that most such measurements were made during the first post-PCI day [23]. The timing of the peak creatinine level was also congruent with that in other previously published studies [25].

PCI-related complications were defined using a composite endpoint that included severe flow-limiting coronary dissection or coronary perforation, myocardial infarction after PCI, cardiogenic shock or heart failure, cerebral bleeding or stroke, and other bleeding complications. Bleeding complications were defined as those requiring transfusion, prolonging hospital stay, and/or causing a decrease in hemoglobin of >3.0 g/dL [26]. When present, bleeding complications were classified using the bleeding site: puncture-site bleeding (including external bleeding or a hematoma diameter >10 cm [femoral], >5 cm [brachial], or >2 cm [radial] at the access site), retroperitoneal bleeding, gastrointestinal bleeding, genitourinary bleeding, or other types of bleeding. This bleeding complication definition is consistent with that for a grade 3 (A–C) bleed, according to the Bleeding Academic Research Consortium [27]. Our definitions of complications were also consistent with the NCDR CathPCI registry; additional information on data elements and definitions is available on their web site: http://www.ncdr.com/webncdr/cathpci/.

Continuous variables are expressed as means and standard deviations or medians (IQR), as appropriate for the data distribution; categorical variables are expressed as percentages. For continuous variables, changes from baseline were evaluated using Student’s *t* test or the Mann-Whitney *U*-test, as appropriate for the data distribution; chi-squared or Fisher’s exact tests were used for assessing changes in categorical variables. Smooth spline plots were used to analyze correlations between BMI and CI-AKI, and between BMI and the V/CrCl ratio. A multivariate logistic regression analysis evaluated the effect of BMI on the incidence of a V/CrCl ratio > 3 (V/CrCl>3). Variables with P-value < 0.25 in the univariate modeling were included in a multivariate logistic regression model to identify factors associated with V/CrCl>3. To assess CI-AKI using the logistic regression model in each BMI group, the following variables were entered: female gender, previous heart failure, diabetes mellitus, peripheral artery disease, hypertension, smoking, previous PCI, heart failure upon admission, cardiogenic shock upon admission, cardiopulmonary arrest upon admission, femoral artery approach, intra-aortic balloon pump, ST-elevation myocardial infarction, non ST-elevation myocardial infarction, unstable angina, type C lesion, use of intravascular ultrasound, BMI, age, and V/CrCl>3.

We performed two sensitivity analyses. The first was a multivariate logistic regression analysis to assess the association of BMI with V/CrCl>3, excluding the 3832 patients with complex lesions (PCI of the left main coronary artery, type C lesion, bifurcation lesion, chronic total occlusion lesion, Rotablator use, multivessel PCI) because lesion complexity may affect CV [28,29]. Second, we analyzed associations with CV, V/CrCl ratio, and BMI for the 3283 patients with a CrCl <60 mL/min (indicative of a high CI-AKI risk) in a subgroup analysis [22,30].

All statistical calculations and analyses were performed using SPSS (version 24, SPSS, Chicago, IL, USA), and R 3.2.3 (R Foundation for Statistical Computing, Vienna, Austria); P-values < 0.05 were considered statistically significant.

## Results

The mean age of the patients in our study group was 68.3 ± 11.2 years; they had a mean BMI of 24.3 ± 3.7 kg/m^2^. Among the study participants, 48.8% presented with acute coronary syndrome. The overall incidence of CI-AKI was 9.5% (n = 838), with an in-hospital mortality rate of 1.7% (n = 153).

The in-hospital outcomes for both BMI groups are summarized in Table 1. The incidences of CI-AKI were comparable between the groups (9.5% for each group). In both BMI groups, significant associations were observed between V/CrCl>3 and CI-AKI in the logistic regression modeling. The odds ratios [ORs] of CI-AKI occurring in the presence of V/CrCl>3 were 1.77 (95% confidence interval [CI], 1.42–2.21; P < 0.001)) for the low-BMI group and 1.67 (95% CI, 1.22–2.29, P = 0.001) for the high-BMI group (Tables 2 and 3). Other clinical factors associated with CI-AKI for the low- and high-BMI groups are also reported in Tables 2 and 3.

**Table 1:** Baseline characteristics and in-hospital outcomes for all patients.

	BMI ≤25 low-BMI group n = 5499 (62.6%)	BMI >25 high-BMI group n = 3283 (37.4%)	P-value
Age, y	70.3 ± 10.5	64.8 ± 11.5	<0.001
Females, n (%)	1252 (22.8)	580 (17.7)	<0.001
BMI (kg/m^2^)	22.1±2.1	27.9±2.8	<0.001
Creatinine value (mg/dL)	0.9 [0.7, 1.1]	0.9 [0.7, 1.1]	0.068
Creatinine clearance (mL/min.)	64.1 ± 26.9	88.7 ± 36.1	<0.001
Creatinine clearance <60 mL/min, n (%)	2578 (46.9)	705 (21.5)	<0.001
Creatinine >1.5 mg/dL	311 (5.7)	176 (5.4)	0.559
Previous myocardial infarction, n (%)	1231 (22.4)	787 (24.0)	0.087
Previous heart failure, n (%)	534 (9.7)	223 (6.8)	<0.001
Diabetes mellitus, n (%)	2144 (39.0)	1568 (47.8)	<0.001
Diabetes mellitus with insulin, n (%)	348 (6.3)	268 (8.2)	0.001
Cerebrovascular disease, n (%)	514 (9.3)	237 (7.2)	0.001
Peripheral artery disease, n (%)	555 (10.1)	212 (6.5)	<0.001
Chronic lung disease, n (%)	214 (3.9)	81 (2.5)	<0.001
Hypertension, n (%)	3909 (71.1)	2610 (79.5)	<0.001
Smoking, n (%)	1697 (30.9)	1293 (39.4)	<0.001
Dyslipidemia, n (%)	3383 (61.5)	2382 (72.6)	<0.001
Previous PCI, n (%)	1913 (34.8)	1280 (39.0)	<0.001
Previous coronary bypass, n (%)	291 (5.3)	138 (4.2)	0.022
Heart failure on admission, n (%)	721 (13.1)	298 (9.1)	<0.001
Cardiogenic shock upon admission, n (%)	253 (4.6)	111 (3.4)	0.006
Cardiopulmonary arrest upon admission, n (%)	144 (2.6)	82 (2.5)	0.729
Puncture site			0.020
Femoral artery approach, n (%)	2848 (51.8)	1599 (48.7)	
Radial artery approach, n (%)	2547 (46.3)	1619 (49.3)	
Brachial artery approach, n (%)	104 (1.9)	65 (2.0)	
Intra-aortic balloon pump, n (%)	410 (7.5)	210 (6.4)	0.061
PCI indication			<0.001
ST-elevation myocardial infarction, n (%)	1424 (25.9)	756 (23.0)	
Non-ST-elevation myocardial infarction, n (%)	473 (8.6)	241 (7.3)	
Unstable angina, n (%)	896 (16.3)	500 (15.2)	
Elective PCI, n (%)	2706 (49.2)	1786 (54.4)	
3-vessel disease, n (%)	1095 (19.9)	647 (19.7)	0.816
PCI artery (%)			
Left main coronary artery, n (%)	277 (5.0)	118 (3.6)	0.002
Left anterior descending artery, n (%)	2818 (51.2)	1575 (48.0)	0.003
Left circumflex artery, n (%)	1184 (21.5)	791 (24.1)	0.005
Right coronary artery, n (%)	1818 (33.1)	1102 (33.6)	0.626
Type C lesion, n (%)	1965 (35.7)	1185 (36.1)	0.733
Bifurcation lesion, n (%)	1853 (33.7)	1055 (32.1)	0.132
Chronic total occlusion lesion, n (%)	381 (6.9)	264 (8.0)	0.053
Balloon angioplasty, n (%)	912 (16.6)	528 (16.1)	0.539
Bare metal stent, n (%)	829 (15.1)	457 (13.9)	0.143
Drug eluting stent, n (%)	4243 (77.2)	2581 (78.6)	0.112
Rotablator use, n (%)	147 (2.7)	80 (2.4)	0.499
Use of intravascular ultrasound, n (%)	4677 (85.1)	2785 (84.8)	0.779
PCI for multi-vessels, n (%)	525 (9.5)	294 9.0)	0.356
Contrast volume (mL)	166 ± 74	174 ± 82	<0.001
V/CrCl>3, n (%)	2051 (37.3)	670 (20.4)	<0.001
In-hospital outcomes			
In-hospital mortality, n (%)	103 (1.9)	50 (1.5)	0.225
All complications, n (%)	511 (9.3)	232 (7.1)	<0.001
Coronary dissection, n (%)	55 (1.0)	23 (0.7)	0.148
Coronary perforation, n (%)	47 (0.9)	30 (0.9)	0.774
Myocardial infarction, n (%)	70 (1.3)	44 (1.3)	0.788
Cardiogenic shock, n (%)	96 (1.7)	51 (1.6)	0.497
Heart failure, n (%)	88 (1.6)	41 (1.2)	0.185
Cerebral infarction, n (%)	21 (0.4)	8 (0.2)	0.275
Intracranial hemorrhage, n (%)	5 (0.09)	2 (0.06)	0.630
Cardiac tamponade, n (%)	20 (0.4)	8 (0.2)	0.334
Hemodialysis, n (%)	59 (1.1)	38 (1.2)	0.714
Transfusion, n	145 (2.6)	50 (1.5)	0.001
Bleeding all, n (%)	163 (3.0)	69 (2.1)	0.015
Puncture site bleeding, n	30 (0.5)	15 (0.5)	0.573
Puncture site hematoma, n (%)	39 (0.7)	13 (0.4)	0.064
Peritoneal bleeding, n (%)	11 (0.2)	2 (0.06)	0.101
Gastrointestinal bleeding, n (%)	19 (0.3)	9 (0.3)	0.566
Genitourinary bleeding, n (%)	3 (0.05)	1 (0.03)	0.609
Other bleeding, n (%)	70 (1.3)	34 (1.0)	0.320
Contrast induced acute kidney injury, n (%)	525 (9.5)	313 (9.5)	0.984

BMI, body mass index; PCI, percutaneous coronary intervention; V/CrCl, contrast volume to creatinine clearance ratio

**Table 2:** A multivariate logistic regression analysis of contrast induced acute kidney injury in the low-BMI group.

	Odds ratio (confidential interval)	P-value
Female	0.91 (0.71–1.15)	0.42
BMI	1.01 (0.96–1.05)	0.83
Previous heart failure	1.54 (1.13–2.11)	0.007
Diabetes mellitus	1.38 (1.13–1.70)	0.002
Peripheral artery disease	1.64 (1.21–2.23)	0.001
Hypertension	1.64 (1.28–2.10)	<0.001
Smoking	1.38 (1.10–1.73)	0.005
Previous PCI	0.74 (0.57–0.95)	0.019
Heart failure at admission	1.72 (1.35–2.20)	<0.001
Cardiogenic shock at admission	1.14 (0.76–1.72)	0.53
Cardiopulmonary arrest at admission	1.36 (0.82–2.26)	0.24
Femoral artery approach	1.60 (1.28–2.01)	<0.001
Intra-aortic balloon pump	2.48 (1.87–3.30)	<0.001
ST-elevation myocardial infarction	4.52 (3.41–5.98)	<0.001
Non-ST-elevation myocardial infarction	2.98 (2.10–4.23)	<0.001
Unstable angina	1.46 (1.03–2.05)	0.032
Type C lesion	1.39 (1.13–1.71)	0.002
Use of intravascular ultrasound	0.85 (0.66–1.09)	0.20
V/CrCl>3	1.77 (1.42–2.21)	<0.001
Age	1.03 (1.02–1.04)	<0.001

BMI, body mass index; PCI, percutaneous coronary intervention; V/CrCl, contrast volume to creatinine clearance ratio

**Table 3:** Multivariate logistic regression analysis of contrast induced acute kidney injury in the high-BMI group.

	Odds ratio (confidential interval)	P-value
Female	0.99 (0.70–1.41)	0.96
BMI	1.01 (0.97–1.06)	0.58
Previous heart failure	1.24 (0.74–2.08)	0.42
Diabetes mellitus	1.19 (0.91–1.55)	0.20
Peripheral artery disease	1.62 (0.98–2.68)	0.062
Hypertension	1.56 (1.10–2.21)	0.013
Smoke	1.06 (0.80–1.42)	0.68
Previous PCI	0.73 (0.52–1.02)	0.066
Heart failure upon admission	1.51 (1.04–2.20)	0.031
Cardiogenic shock upon admission	1.20 (0.66–2.19)	0.54
Cardiopulmonary arrest upon admission	3.40 (1.79–6.45)	<0.001
Femoral artery approach	1.34 (1.01–1.78)	0.042
Intra-aortic balloon pump	3.39 (2.30–5.00)	<0.001
ST-elevation myocardial infarction	4.55 (3.17–6.55)	<0.001
Non-ST-elevation myocardial infarction	2.95 (1.84–4.73)	<0.001
Unstable angina	1.40 (0.90–2.78)	0.14
Type C lesion	0.90 (0.68–1.20)	0.48
Use of intravascular ultrasound	0.86 (0.61–1.20)	0.37
V/CrCl>3	1.67 (1.22–2.29)	0.001
Age	1.02 (1.01–1.04)	<0.001

BMI, body mass index; PCI, percutaneous coronary intervention; V/CrCl, contrast volume to creatinine clearance ratio

**Fig 2:**
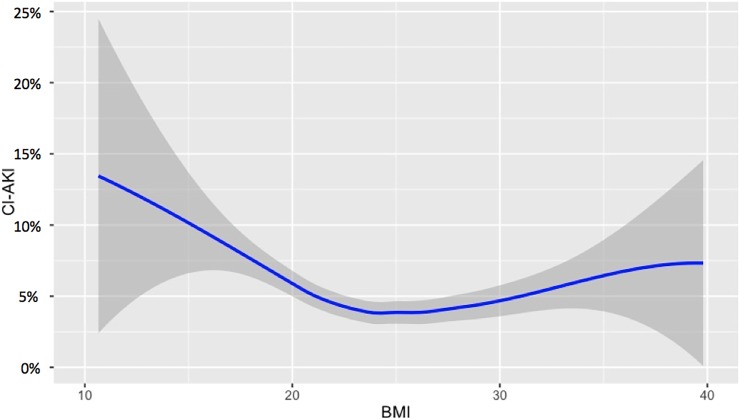
Smooth spline plots of BMIs and CI-AKI incidence rates. The smooth spline is shown as a blue line and the gray area represents the 95% confidence intervals. BMI, body mass index; CI-AKI, contrast-induced acute kidney injury.

**Fig 3:**
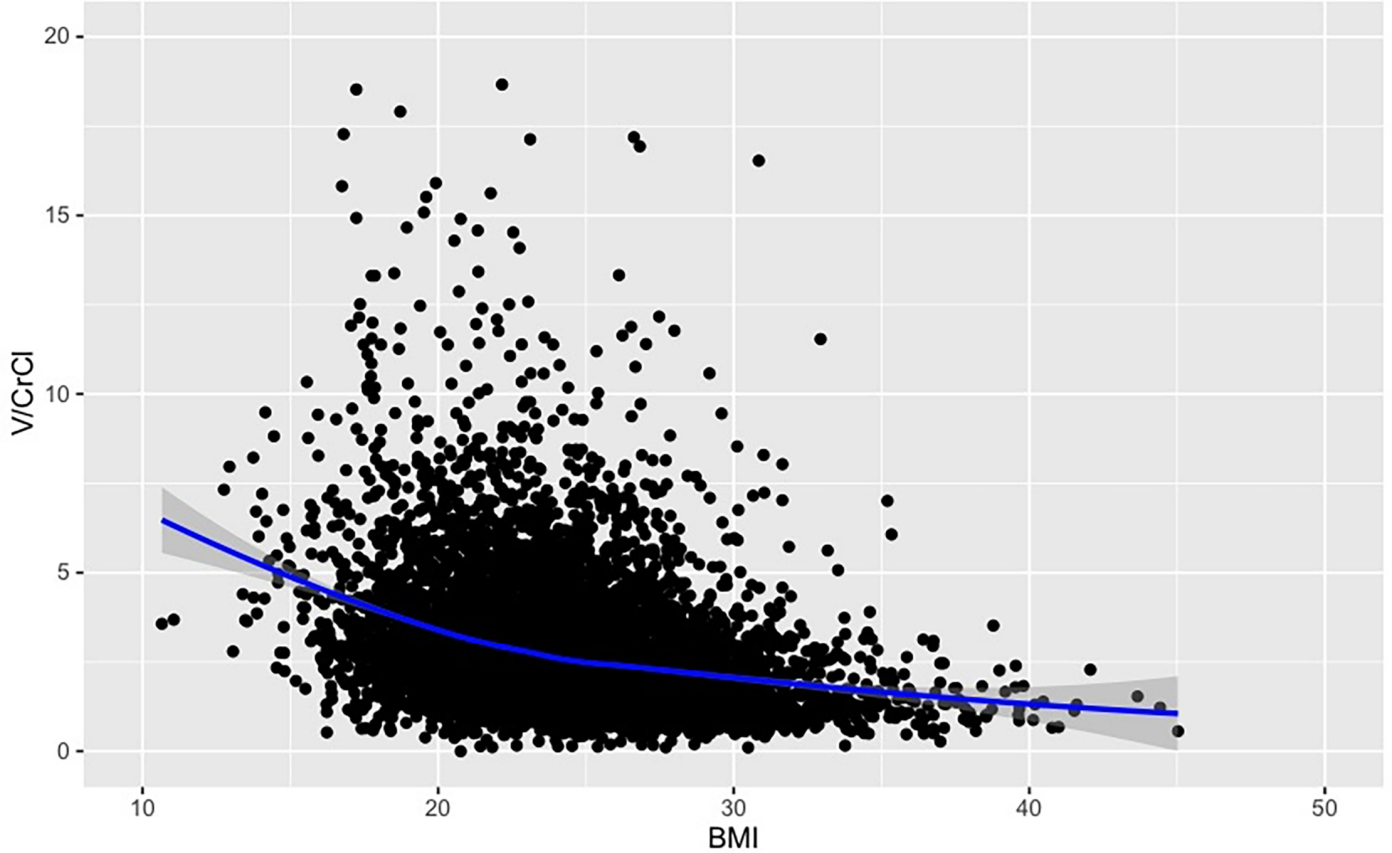
Smooth spline plots of BMIs and V/CrCl ratios. V/CrCl ratios are plotted, with a smooth spline shown as a blue line. The gray area represents the 95% confidence intervals. BMI, body mass index; V/CrCl, contrast volume to creatinine clearance ratio.

The baseline characteristics of patients in the low- and high-BMI groups, including procedural information, are summarized in Table 1. The BMI and CI-AKI incidence data demonstrated a reverse J-curve relationship (Fig 2). Overall, the low-BMI group included older patients and had a higher proportion of females than did the high-BMI group. Importantly, the proportion of patients with renal dysfunction (CrCl <60 mL/min.) was also higher in the low-BMI group than in the high-BMI group (46.9% vs. 21.5%, P < 0.001), although the creatinine values were similar between the two groups (low-BMI group, 0.9 [0.7, 1.1] mg/dL; high-BMI group, 0.9 [0.7, 1.1] mg/dL, P = 0.068). Additionally, the proportion of patients with a V/CrCl>3 was higher in the low-BMI (37.3%) group than in the high-BMI (20.4%) group (P < 0.001) and the V/CrCl ratios were significantly different between the groups (low-BMI, 2.5 [1.8, 3.6]; high-BMI, 1.9 [1.3 vs. 2.8]; P < 0.001). The smooth spline plots showed the inverse relationship between the V/CrCl ratio and BMI (Fig 3).

**Fig 4:**
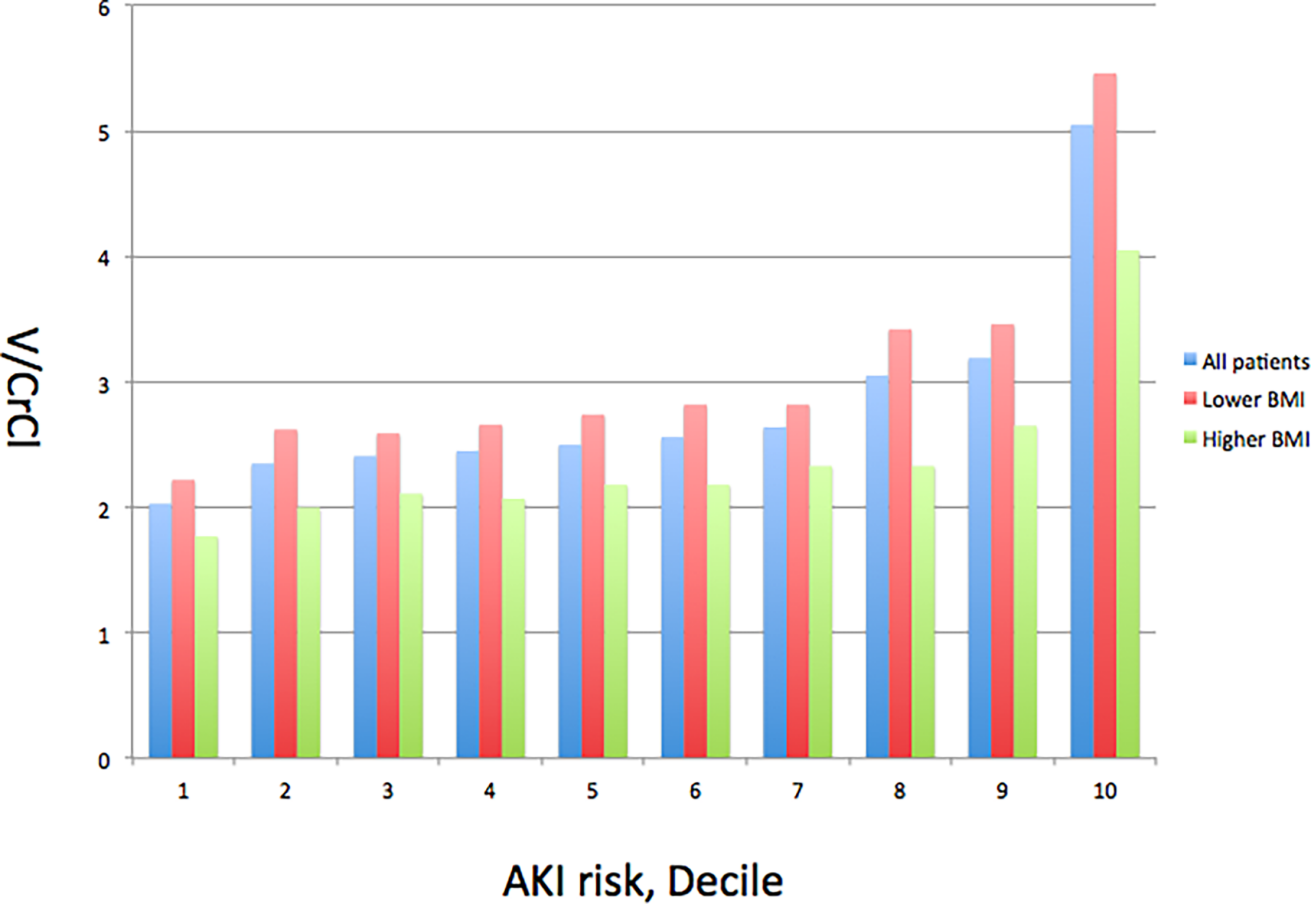
Mean V/CrCl ratio compared to AKI risk decile for all patients, the low-BMI group, and the high-BMI group. V/CrCl, contrast volume to creatinine clearance ratio; AKI, acute kidney injury; BMI, body mass index.

Since the smooth spline plots revealed higher V/CrCl ratios in patients in the low-BMI group, we sought to identify the effect of BMI on the incidence of V/CrCl>3 observations. Using a multivariate logistic regression analysis, a low BMI was as a significant predictor of a V/CrCl>3 (OR, for 1 unit decrease in BMI, 1.08; 95% CI, 1.05–1.10; P < 0.001; Table 4). In the all-patient analysis of CI-AKI risk and V/CrCl ratio, patients with a higher CI-AKI risk tended to have higher V/CrCl ratios, particularly those in the low-BMI group (Fig 4).

**Table 4:** Multivariate logistic regression analysis for V/CrCl>3.

	Multivariate analysis	
Variable	Odds ratio (confidence interval)	P value
Age	1.05 (1.04–1.06)	<0.001
Female	1.36 (1.19–1.55)	<0.001
Creatinine clearance <60 mL/min	4.30 (3.78–4.90)	<0.001
BMI (one unit decrement)	1.08 (1.05–1.10)	<0.001
Previous myocardial infarction	1.19 (1.02–1.38)	0.026
Previous heart failure	0.98 (0.81–1.19)	0.83
Cerebrovascular disease	0.91 (0.75–1.09)	0.30
Peripheral artery disease	1.32 (1.10–1.58)	0.003
Chronic lung disease	1.11 (0.84–1.46)	0.48
Hypertension	1.39 (1.21–1.59)	<0.001
Smoking	1.12 (0.98–1.27)	0.093
Dyslipidemia	1.02 (0.91–1.16)	0.70
Previous PCI	0.84 (0.73–0.97)	0.016
Previous coronary bypass	1.37 (1.07–1.74)	0.012
Heart failure upon admission	1.22 (1.03–1.45)	0.025
Cardiogenic shock upon admission	1.06 (0.75–1.49)	0.75
Cardiopulmonary arrest upon admission	1.16 (0.77–1.76)	0.49
Femoral artery approach	2.19 (1.95–2.47)	<0.001
Intra-aortic balloon pump	1.67 (1.32–2.11)	<0.001
ST-elevation myocardial infarction	0.89 (0.76–1.05)	0.160
Non-ST-elevation myocardial infarction	0.97 (0.78–1.20)	0.77
Unstable angina	1.22 (1.04–1.43)	0.015
3-vessel disease	1.13 (0.98–1.29)	0.093
PCI of the left main coronary artery	1.06 (0.81–1.38)	0.68
PCI of the left anterior descending artery	1.21 (1.07–1.38)	0.003
PCI of the left circumflex artery	1.15 (0.99–1.34)	0.070
Type C lesion	1.21 (1.07–1.37)	0.003
Bifurcation lesion	1.36 (1.20–1.54)	<0.001
Chronic total occlusion lesion	2.79 (2.25–3.46)	<0.001
Rotablator use	1.59 (1.15–2.19)	0.005
Multi-vessel PCI	1.69 (1.38–2.07)	<0.001

V/CrCl, contrast volume to creatinine clearance ratio; BMI, body mass index; PCI, percutaneous coronary intervention

In a sensitivity analysis of patients, excluding those with complex lesions, the BMI tended to remain predictive of V/CrCl>3 (OR, for 1 unit decrease in BMI, 1.04; 95% CI, 1.01–1.07; P = 0.07). In a second sensitivity analysis of patients with CrCl <60 mL/min, the CV administered in each group was similar (low-BMI group vs. high-BMI group, 155 ± 73 mL vs. 154 ± 82 mL; P = 0.72), whereas the V/CrCl ratios were quite different between the groups (3.4 [2.5, 4.8] vs. 3.0 [2.2, 4.2], respectively, P < 0.001). After performing a multivariate logistic regression analysis for this subgroup, BMI remained a significant predictor of V/CrCl>3 (OR, for 1 unit decrease in BMI, 1.07; 95% CI, 1.05–1.10; P < 0.001) risk.

## Discussion

The major findings from our study are as follows. First, V/CrCl>3 was strongly associated with CI-AKI incidence, regardless of patient BMI. Second, the relationship between BMI and CI-AKI incidence demonstrated a reverse J-curve relationship. Third, physicians were noted to tend toward the use of higher V/CrCl ratios in lean patients, particularly in those with a higher CI-AKI; physicians appear to focus on the absolute volume of contrast used, not on the V/CrCl ratio. Greater consideration of these factors will allow physicians to identify patients who are most at risk for CI-AKI and, therefore, limit the CV used during PCI procedures, especially in lean patients.

CI-AKI after PCI remains a major post-procedural complication due to its significant association with mortality, particularly among low-BMI patients [1,2,31]. However, providing adequate hydration and minimizing CVs are proven strategies for lowering the CI-AKI risk. In our study, a reverse J-curve relationship was observed between BMI and CI-AKI incidence. Hence, this identification of the effects of BMI on the incidence of CI-AKI may contribute to the ability of physicians to prevent CI-AKI. Conventionally, physicians often try to use the least amount of contrast possible, but higher volumes may be required in more complex cases. To eliminate the effects of these confounders, in the present study, we performed multivariate logistic regression analyses to assess the predictors of V/CrCl>3, including complex treatment scenarios such as PCI of the left main coronary artery, type C lesions, bifurcations, chronic total occlusions, Rotablator use, and multivessel PCIs. Moreover, we excluded these complex cases when conducting a sensitivity analysis. The results demonstrated that physicians tend to use higher V/CrCl ratios in lean patients [29]. Since a V/CrCl>3 was strongly associated with CI-AKI in the low-BMI group, in our study, physicians need to recognize the role of CrCl values and attempt more rigorous intra-procedural CV reductions in lean patients; the use of biplane angiography, careful minimization of contrast injection, and the use of intravascular ultrasound guidance should be considered [7,32].

To distinguish between the high- and low-BMI groups, in this study, the BMI cut off was set at 25 kg/m^2^ because the mean BMI for Asian patients undergoing PCI is approximately 25 kg/m^2^, lower than for Western populations [33,34]. However, the obesity paradox has been observed in both Asian and Western patients undergoing PCI [34,35]. In addition, we showed that the relationship between BMI and CI-AKI incidence follows a reverse J-curve relationship. Because the number of elderly people undergoing PCI is increasing, and the average body habitus is getting leaner in aging societies [36,37], a focus on relatively lean patients (BMI <25 kg/m^2^) is needed to improve in-hospital outcomes. Since we observed a tendency for physicians to use higher V/CrCls in lean patients in the present real-world registry, the need for high CVs, relative to patient CrCl, should be carefully considered.

Previously, Amin et al. showed that the administered CVs were not different across patients, despite variations in CI-AKI risk. This was presumed to be because physicians focus on absolute CVs and not on the V/CrCl ratio [28]. We believe that this is the reason why V/CrCl ratios < 3 were not achieved, especially in lean patients. We also demonstrated that patients with a higher CI-AKI risk tend to have higher V/CrCl ratios, especially patients with a low BMI; this is contrary to the guideline recommendation [4]. The V/CrCl ratio is a well-established and internationally validated index, developed to decrease the risk of CI-AKI (with a target < 3) [5,7]. However, the actual CV used during PCI procedures differs among currently practicing physicians [28]. This study, in addition to improving the ability of physicians to recognize patients with higher CI-AKI risks, provides physicians with feedback regarding the value of this index from a large-scale, national registry data investigation [20,28,38].

We also showed a higher incidence of CI-AKI in obese patients, based on the reverse J-curve relationship between BMI and CI-AKI incidence. Buschur et al. [39] found that poor fluoroscopic visualization and miscalculation of renal function is common in obese patients. We believe that a vulnerability to contrast media and an erroneous estimation of renal function are responsible for this phenomenon. The greater vulnerability to contrast media, among obese individuals, might be attributed to hyperleptinemia, increased sympathetic activity, hypoadiponectinemia, and high levels of free fatty acids and endothelin-1 [40].

There are several limitations in our study. First, this was a prospective, observational study. The non-randomized of the study; the effects of unmeasured confounders; and the potential for selection bias and surgeon bias to exist, even after adjustment, might have influenced the multivariate analysis outcomes. Second, although the registry includes a relatively large number of PCI procedures, performed at multiple centers, not all hospitals in Japan participate in the registry. Further, not all participating hospitals were fully investigated throughout the study period. However, the characteristics of the patients in our registry were similar to those in the national PCI registry [23,41]. Further, compared to the NCDR registry [19], the patients in our registry were older, included a higher proportion of males, and had a higher overall proportion of individuals with diabetes, a positive smoking status, and more complex lesions. Despite these differences, the mortality risk and CI-AKI models derived from the NCDR registry were able to be correctly applied to our registry [23]. Therefore, we believe that our registry is one of the most representative PCI databases in Japan. Further, our results provide the most complete assessment of current practice patterns in Japan, and the results may be applied globally. Third, we excluded patients with missing baseline and post-procedural BMI, creatinine, and CV values; the data for the missing patients are included in S1 and S2 Tables. Since creatinine values were not consistently collected from stable patients, missing values were more often associated with these patients; thus, these exclusions may have biased our results. Fourth, the causes of post-PCI CI-AKI are multifactorial [42], and the use of contrast agents is only one of the causes. We also identified acute coronary syndrome and the use of intra-aortic balloon pumps as additional risk factors associated with the development of CI-AKI. Finally, BMI might be a confounder of the relationship between the V/CrCl ratio and CI-AKI incidence because the low-BMI patients had low CrCl values, as shown in our study. Patients with low body weights also tend to have higher V/CrCl ratios. However, BMI is not equivalent to body weight, and the association of BMI with CrCl is complex because it does not demonstrate a positive correlation after adjustment for total body surface area [43]. Moreover, CrCl was estimated, using the Cockcroft-Gault equation, to be higher in lean patients than in other patients because of their lower muscle mass; this indicates that BMI and CrCl are not positively correlated [44]. Because of the complicated relationship between BMI and CrCl, the ability to demonstrate an association between the V/CrCl ratio and BMI is valuable.

In conclusion, a V/CrCl>3 was associated with CI-AKI in patients with low (≤25 kg/m^2^) and high (>25 kg/m^2^) BMIs, and the relationship between BMI and CI-AKI incidence followed a reverse J-curve relationship. Importantly, we identified a tendency for physicians to use high V/CrCl ratios in patients with low BMIs, particularly in those with higher CI-AKI risk. This is believed to occur because physicians focus on the absolute amount of CV used, rather than on the V/CrCl ratio. Furthermore, high V/CrCl ratios in low-BMI patients remained unchanged after we excluded complex cases. The identification of the BMI effects on CI-AKI incidence might improve the ability of physicians to prevent CI-AKI by selecting more optimal CVs for each patient.

## Supporting information

S1 Table Baseline characteristics all records excluding patients on dialysis. (DOCX)

S2 Table In-hospital outcomes of all records excluding patients on dialysis. (DOCX)
