# Supplementary material for: Effects of body habitus on contrast-induced acute kidney injury after percutaneous coronary intervention
Source: PLoS One. 2018 Sep 13;13(9):e0203352. doi: 10.1371/journal.pone.0203352 (PMC6136739; doi:10.1371/journal.pone.0203352)
Supplement: S1 Table — (DOCX) [file pone.0203352.s001.docx]

S1 Table: Baseline characteristics all records excluding patients on dialysis

|  | Patients on this study  % (n=8782) | Patients with missing values  % (n=3172) | P value |
| --- | --- | --- | --- |
| Age | 68.3±11.2 | 68.7±10.9 | 0.067 |
| Female | 20.8% (1832) | 19.4% (489) | 0.099 |
| BMI | 24.2±3.7 | 24.5±3.5 | 0.001 |
| Previous myocardial infarction | 23.0% (2018) | 28.5% (716) | <0.001 |
| Previous heart failure | 8.6% (757) | 10.1% (250) | 0.023 |
| Diabetes mellitus | 42.2% (3712) | 43.0% (1063) | 0.525 |
| Diabetes mellitus  with insulin | 7.0% (616) | 8.2% (204) | 0.037 |
| Cerebrovascular disease | 8.6% (751) | 8.7% (216) | 0.804 |
| Peripheral artery disease | 8.7% (767) | 8.5% (211) | 0.720 |
| Chronic lung disease | 3.4% (295) | 2.8% (70) | 0.186 |
| Hypertension | 74.2% (6519) | 78.9% (1950) | <0.001 |
| Smoking | 34.0% (2990) | 30.6% (754) | 0.001 |
| Dyslipidemia | 65.6% (5765) | 71.0% (1744) | <0.001 |
| Previous PCI | 36.4% (3193) | 49.1% (1255) | <0.001 |
| Previous coronary bypass | 4.9% (429) | 5.0% (127) | 0.866 |
| Heart failure on admission | 11.6% (1019) | 12.0% (377) | 0.550 |
| Cardiogenic shock on admission | 4.1% (364) | 4.5% (140) | 0.450 |
| Cardiopulmonary arrest  on admission | 2.6% (226) | 3.0% (93) | 0.247 |
| Puncture site |  |  | <0.001 |
| Femoral artery approach | 50.6% (4447) | 45.7% (1429) |  |
| Radial artery approach | 47.4% (4166) | 51.7% (1616) |  |
| Brachial artery approach | 1.9% (169) | 2.7% (83) |  |
| Intra aortic balloon pump | 7.0% (619) | 7.2% (225) | 0.811 |
| PCI indications |  |  | <0.001 |
| ST-elevation  myocardial infarction | 24.8% (2178) | 12.2% (374) |  |
| Non ST-elevation  myocardial infarction | 8.1% (712) | 3.6% (109) |  |
| Unstable angina | 15.9% (1396) | 9.8% (300) |  |
| Elective PCI | 51.2% (4495) | 74.3% (2264) |  |
| PCI performed artery |  |  |  |
| Left main | 4.5% (395) | 5.1% (162) | 0.163 |
| Left anterior descending artery | 50.0% (4393) | 45.5% (1443) | <0.001 |
| Left circumflex artery | 22.4% (1975) | 24.9% (789) | 0.006 |
| Right coronary artery | 33.2% (2920) | 32.4% (1027) | 0.370 |
| Balloon angioplasty | 16.4% (1440) | 17.8% (566) | 0.062 |
| Bare metal stent | 14.6% (1286) | 11.2% (355) | <0.001 |
| Drug eluting stent | 77.7% (6824) | 75.6% (2398) | 0.015 |
| Rotablator use | 2.6% (227) | 3.4% (109) | 0.013 |
| Intravascular ultrasound use | 85.0% (7462) | 81.3% (2580) | <0.001 |
| PCI for multi-vessels | 9.3% (819) | 11.1% (337) | 0.006 |

BMI: body mass index, PCI: percutaneous coronary intervention
