# Supplementary material for: Effects of body habitus on contrast-induced acute kidney injury after percutaneous coronary intervention
Source: PLoS One. 2018 Sep 13;13(9):e0203352. doi: 10.1371/journal.pone.0203352 (PMC6136739; doi:10.1371/journal.pone.0203352)
Supplement: S2 Table — (DOCX) [file pone.0203352.s002.docx]

S2 Table : In-hospital outcomes of all records excluding patients on dialysis

|  | Patients on this study  % (n=8782) | Patients with missing values  % (n=3172) | P value |
| --- | --- | --- | --- |
| All complications | 8.4% (743) | 7.4% (227) | 0.074 |
| In-hospital mortality | 1.7% (153) | 3.3% (104) | <0.001 |
| Coronary Dissection | 0.9% (78) | 0.9% (27) | 0.981 |
| Coronary Perforation | 0.9% (77) | 0.8% (24) | 0.637 |
| Myocardial infarction | 1.3% (114) | 1.3% (40) | 0.926 |
| Cardiogenic shock | 1.7% (147) | 1.9% (57) | 0.484 |
| Heart failure | 1.5% (129) | 1.4% (43) | 0.841 |
| Cerebral infarction | 0.3% (29) | 0.3% (9) | 0.761 |
| Intracranial hemorrhage | 0.08% (7) | 0.03% (1) | 0.389 |
| Cardiac tamponade | 0.3% (28) | 0.2% (5) | 0.161 |
| Hemodialysis | 1.1% (97) | 0.7% (20) | 0.030 |
| Transfusion | 2.2% (195) | 2.7% (82) | 0.162 |
| Bleeding all | 2.6% (232) | 2.0% (61) | 0.048 |
| Puncture site bleeding | 0.5% (45) | 0.5% (15) | 0.876 |
| Puncture site hematoma | 0.6% (52) | 0.3% (10) | 0.079 |
| Peritoneal bleeding | 0.1% (13) | 0.03% (1) | 0.109 |
| Gastrointestinal bleeding | 0.3% (28) | 0.2% (6) | 0.312 |
| Genitourinary bleeding | 0.05% (4) | 0.07% (2) | 0.677 |
| Other bleeding | 1.2% (104) | 1.0% (31) | 0.436 |
